# Synthetic-Type Control Charts for Time-Between-Events Monitoring

**DOI:** 10.1371/journal.pone.0065440

**Published:** 2013-06-03

**Authors:** Fang Yen Yen, Khoo Michael Boon Chong, Lee Ming Ha

**Affiliations:** 1 School of Mathematical Sciences, Universiti Sains Malaysia, Penang, Malaysia; 2 School of Engineering, Computing and Science, Swinburne University of Technology, Sarawak Campus, Kuching, Sarawak, Malaysia; Universidad de Valladolid, Spain

## Abstract

This paper proposes three synthetic-type control charts to monitor the mean time-between-events of a homogenous Poisson process. The first proposed chart combines an Erlang (cumulative time between events, *T_r_*) chart and a conforming run length (CRL) chart, denoted as Synth-*T_r_* chart. The second proposed chart combines an exponential (or *T*) chart and a group conforming run length (GCRL) chart, denoted as GR-*T* chart. The third proposed chart combines an Erlang chart and a GCRL chart, denoted as GR-*T_r_* chart. By using a Markov chain approach, the zero- and steady-state average number of observations to signal (ANOS) of the proposed charts are obtained, in order to evaluate the performance of the three charts. The optimal design of the proposed charts is shown in this paper. The proposed charts are superior to the existing *T* chart, *T_r_* chart, and Synth-*T* chart. As compared to the EWMA-*T* chart, the GR-*T* chart performs better in detecting large shifts, in terms of the zero- and steady-state performances. The zero-state Synth-*T_4_* and GR-*T_r_* (*r* = 3 or 4) charts outperform the EWMA-*T* chart for all shifts, whereas the Synth-*T_r_* (*r* = 2 or 3) and GR-*T*_2_ charts perform better for moderate to large shifts. For the steady-state process, the Synth-*T_r_* and GR-*T_r_* charts are more efficient than the EWMA-*T* chart in detecting small to moderate shifts.

## Introduction

A control chart is one of the important statistical tools in process quality control to distinguish between the variation due to natural cause and assignable cause. Traditionally, control charts with the three-sigma limits, such as the Shewhart-type 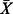 chart, *R* chart, *S* chart, and so on were developed to monitor process quality. Most of these traditional charts are based on the normality assumption or approximation of the underlying process distribution [1]. However, the development of modern technology and process improvement has led to zero-defects or high quality processes, where the defect rate could be very small, up to parts per million [2], [3]. Traditional control charts are not suitable for monitoring such high quality processes since these charts will face certain practical problems including meaningless control limits, higher false alarm rates and failures in detecting process improvement [3], [4].

Due to the inadequacies of traditional control charts, time-between-events (TBE) charts were introduced as alternatives for monitoring high quality processes when non-conforming items are rarely observed. Instead of monitoring the number or proportion of events occurring in a certain sampling interval, the TBE charts monitor the time between successive occurrences of events. The term “events” may refer to the occurrences of non-conforming items in a manufacturing process [3], failure in reliability analysis [5], [6], [7], disease in healthcare management [8], accidents [9], arrival of a customer, etc. The term “time” may also refer to other variables (discrete-type or continuous-type) that measure the quantity observed between the occurrences of events [2]. Therefore, the TBE charts can be used for monitoring any processes with TBE or inter-arrival time random variable. These includes time between component failures in maintenance monitoring [6], time between medical errors [10], time between two consecutive radiation pulses [11], time between asthma attacks [12] and so on [8], [13], [14], [15].

The TBE chart was first developed by Calvin [3] and was further studied by Goh [4], as cumulative count of conforming (CCC) chart based on probability limits. Since then, the conforming run length (CRL) control chart was proposed and has been studied for better interpretation purposes [16], [17]. The CRL chart is also known as the geometric chart [18]. As an extension to the CCC chart, the CCC-*r* chart (or negative binomial chart) was developed to increase the sensitivity in detecting shifts [19], [20]. Radaelli [15] provided a general unified strategy for planning the Shewhart-type TBE chart to monitor the arrival time between successive counts.

Chan et al. [21] proposed a continuous counterpart of the CCC chart called the Cumulative Quantity Control (CQC) chart for monitoring continuous TBE data. It is assumed that the occurrence of an event (i.e. non-conforming item) is modeled by a homogenous Poisson process with a constant rate or mean. Then the observed TBE follows an exponential distribution and the suggested chart is the *t*-chart, exponential chart or *T* chart [5], [22]. Xie et al. [5] extended the CQC chart to the CQC-*r* chart, called the *t_r_*-chart or *T_r_* chart. The CQC-*r* chart is based on the Erlang or Gamma distribution which monitors the cumulative quantity until the *r* ^th^ event is observed. The CQC-*r* chart is named as the gamma chart by Zhang et al. [23].

Besides the TBE charts based on probability limits, the TBE charts based on the CUSUM or EWMA method have also been proposed to monitor both variables and attributes TBE data. These include the Poisson CUSUM and exponential CUSUM or *t*-CUSUM charts [24], [25]; geometric CUSUM and geometric EWMA charts [26], [27], [28]; exponential EWMA or EWMA-*T* chart [29], exponential EWMA chart with estimated parameter [30] and so on. The robustness studies of the exponential CUSUM and exponential EWMA charts showed that these charts can be designed to be extremely robust to departures from the assumed distribution [31], [32].

Comparisons of the CQC, CQC-*r*, exponential CUSUM, as well as the exponential EWMA charts have been carried out by Liu et al. [33], Sharma et al. [34] and Liu et al. [35]. It was concluded that in detecting a large shift, the CQC-*r* chart can be considered, and for a quick detection of small to moderate shifts, the exponential EWMA or exponential CUSUM is superior to both the CQC and CQC-*r* charts. The exponential EWMA and CUSUM charts have quite similar performances.

Other types of TBE charts have also been proposed. For example, Qu et al. [22] integrated the *T* and *T*-CUSUM charts to monitor the exponentially distributed TBE data; a synthetic chart for exponential data was proposed by Scariano & Calzada [36]; the economic designs of the exponential chart and Gamma chart were also suggested [37], [38], [39]; while Jones & Champ [40] studied the phase I TBE chart. Although the TBE charts were initially developed for high quality process monitoring, it can be used also for moderate defective rate processes, provided that the data collected are of the TBE-type. Other advantages of a TBE chart include its ability to detect process improvement, it does not require rational subgroup of samples and it is applicable for any sample size. Those continuous-type TBE charts discussed so far are based on the exponential or gamma distribution, which is the focus of this paper.

A 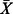 chart in combining with a CRL chart (a discrete-type TBE chart), called a synthetic chart was proposed by Wu & Spedding [41]. Davis & Woodall [42] derived a Markov chain approach for the calculation of the zero- and steady-state (average run length) ARL performance of the synthetic 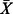 chart. It was shown that the synthetic chart performs better than its counterpart, the Shewhart 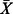 chart. However, the EWMA and CUSUM charts are still superior to the synthetic chart in detecting small to moderate shifts. The synthetic chart has also been studied by others for monitoring attribute data (such as by combining a *np* chart and a CRL chart) and multivariate data (such as by combining a Hotelling's *T* ^2^ chart and a CRL chart) [43], [44], [45], [46]. The optimal statistical and economic designs of the synthetic chart have also been developed [47], [48].

As an extension to the synthetic chart, Gadre & Rattihalli [49] proposed the group runs (GR) chart, which consists of a combination of a 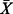 chart and an extended version of CRL chart. The GR chart is also a synthetic-type chart. In this research, we name the extended version of the CRL chart as the group conforming run length chart (GCRL). Basically, the GCRL chart is similar to the CRL chart, except for the decision making procedure. The performance of the GR chart is better than the synthetic 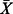 chart, as well as the Shewhart 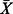 chart.

The synthetic chart based on the exponential data proposed by Scariano & Calzada [36] combines a *T* chart and a CRL chart. In this study, we refer to this chart as the Synth-*T* chart. The zero-state ARL performance of the Synth-*T* chart was studied using a direct formulation method. It has been shown that the zero-state Synth-*T* chart outperforms the standard *T* chart, whereas the exponential EWMA and CUSUM charts are still superior to the Synth-*T* chart, except for very large shifts.

From the literatures, it can be concluded that, the *T_r_* and Synth-*T* charts perform better than the *T* chart. However, the Synth-*T* chart is not effective than the EWMA-*T* chart in detecting almost all shifts, while the *T_r_* chart has a better performance than the EWMA*-T* in detecting large shifts, especially when *r* is larger (i.e.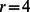). Thus, for a quicker detection of the mean TBE shifts, we propose a type of synthetic chart, called the Synth-*T_r_* chart which combines a *T_r_* chart and a CRL chart. Furthermore, a better performance of the GR chart as compared to the synthetic chart has motivated us to integrate the GR scheme and the TBE chart. Thus, two other types of synthetic charts, called the GR*-T* chart (combined *T* chart and GCRL chart) and GR*-T_r_* chart (combined *T_r_* chart and GCRL chart) are proposed in this paper as well. From an overall perspective, the proposed charts are expected to outperform the existing *T*, *T*_r_, Synth-*T* and EWMA-*T* charts.

Although the idea of combining different control charts, like combining 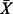 chart and CRL chart to form the synthetic 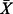 chart, combining *T* ^2^ chart and CRL chart to form the synthetic *T* ^2^ chart, and so on exist in the literature, it should be pointed out that the work in this paper of combining different types of control charts (i.e. *T_r_* chart and CRL chart to form the Synth-*T_r_* chart, *T* chart and GCRL chart to form the GR-*T* chart, *T_r_* chart and GCRL chart to form the GR-*T_r_* chart) is not yet in existence in the literature.

The next section gives a brief overview of the *T*, *T*_r_, Synth*-T* and EWMA-*T* charts. In Section 3, the implementation procedures of the proposed charts are given. The performance evaluation of the charts, based on the average number of observations to signal (ANOS) is derived using the Markov chain approach. An optimal design based on ANOS is explained. This is followed by a comparative study of the proposed charts and the *T*, *T*_r_, Synth-*T* and EWMA-*T* charts in Section 4. It was found that overall the proposed charts increase the speed of mean shift detection, especially the GR-*T_r_* chart. A discussion on the results obtained is given in the same section. Section 5 illustrates the implementation of the proposed charts with three examples. Finally, conclusions are drawn in Section 6 based on the findings of this study.

## Literature Review: A Review on Time-Between-Events Control Charts

Assume that a homogenous Poisson process with events occurrence rate of 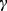 is being monitored. The time-between-events (TBE), 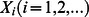 is an independently and identically distributed exponential random variable with cumulative distribution function (cdf) [21]

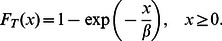
(1)


Here, 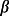 is the mean TBE, which is a reciprocal of the events occurrence rate 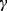 such that 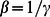. For detecting process deterioration or decreases in the mean TBE (increases in the Poisson rate of occurrences of events), lower-sided control charts are presented in this article.

### The *T* chart and *T_r_* chart

In the monitoring of the exponential TBE (time until an event, *X*) based on probability control limits in Equation (1), the lower-sided *T* chart has the centre line (CL*_T_*) and lower control limit (LCL*_T_*) as follows [21]:
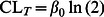
(2)

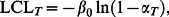
(3)where 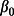 is the in-control mean TBE. Note that CL*_T_* is independent of the given acceptable Type-I error probability, 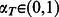.

The *T_r_* chart, based on the Erlang distribution was proposed in order to increase the sensitivity of the *T* chart. When the TBE is modeled by the exponential distribution, the cumulative of a fixed number, *r* of the TBE (time until the *r* ^th^ event, *Y_r_*, in a Poisson process) follows the Erlang distribution with cdf [5]

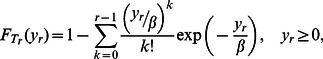
(4)where *β* and *r* are the parameters of the Erlang distribution and 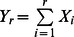. For a given Type-I error probability 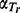, the centre line (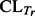) and lower control limit (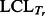) of the *T_r_* chart are easily obtained by solving the following equations [5]:



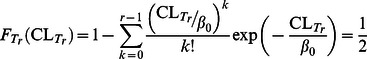
(5)

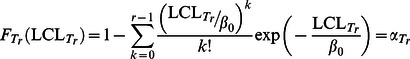
(6)


### The EWMA-T (exponential EWMA) Chart

The statistics for the lower-sided EWMA-*T* chart is [29]

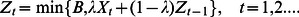
(7)


Here, *B* is a positive boundary and 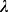 is a smoothing constant such that 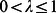. The starting value 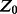 is usually chosen as the process target, 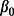 which is the in-control mean TBE. The centre line (CL*_E−T_*) and lower control limit (LCL*_E−T_*) of the EWMA-*T* chart are [50].
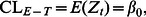
(8)

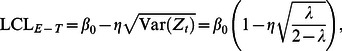
(9)where 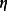 is the parameter that determines the width of the lower limit. The process is assumed to be out-of-control if 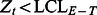. The boundary *B* in Equation (7) is set to be 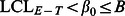 to ensure that the 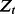 is at most a certain distance away from the LCL*_E−T_*. In this study, 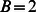 is selected (when 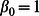) for a better overall performance [29], [30], [31]. Performance analysis based on ANOS using the Markov chain approach of Brook & Evans [51] is adopted in this comparative study. The ANOS derivation based on the Markov chain method can be found in the Appendix S1.

### The Synth-*T* chart

The synthetic chart based on the exponential data instead of the normal distributed data has been developed by Scariano & Calzada [36]. It is a combination of a lower-sided Shewhart individual sub-chart and a CRL sub-chart. The lower-sided Shewhart individual sub-chart is also the *T* chart of Chan et al. [21] and the CRL sub-chart is that of Bourke [17]. In this study, we denote the chart as the Synth-*T* chart which consists of a *T/S* sub-chart and a CRL*/S* sub-chart.

#### *T*/*S* sub-chart

As described in Section 2.1, the TBE, *X* follows an exponential distribution. Similar to Equation (3), the *T/S* sub-chart has the lower control limit
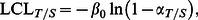
(10)where 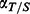 is the Type-I error probability of the *T*/*S* sub-chart. The TBE observation *X* is said to be non-conforming (instead of out-of-control) when 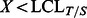.

#### The CRL/*S* sub-chart

**Figure 1:**
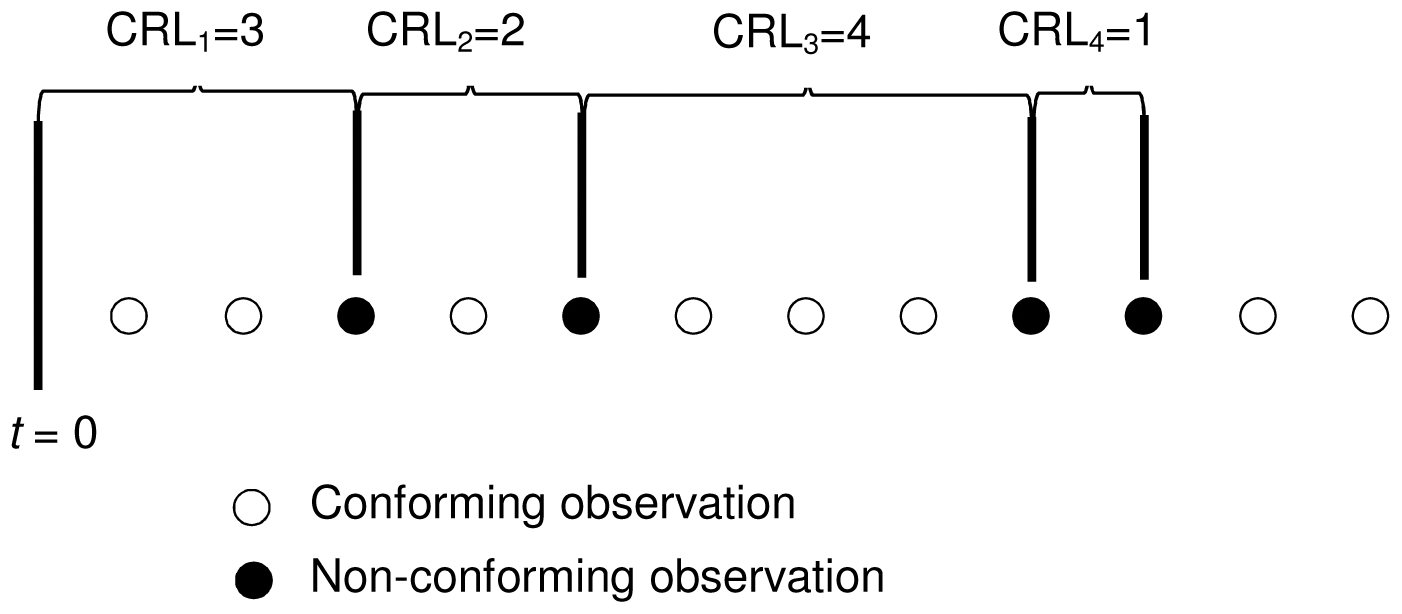
Conforming run length.

The random variable CRL in the CRL/*S* sub-chart counts the number of conforming TBE observations *X′*s between two consecutive non-conforming ones, including the ending non-conforming TBE observation *X*, from the *T*/*S* sub-chart. The CRL follows a geometric distribution with cdf [41]

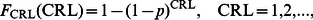
(11)where *p* is the probability of a non-conforming TBE, *X* on the *T/S* sub-chart, such that
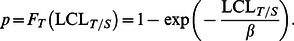
(12)Since the detection of an increase in p is the only concern, the lower control limit of the CRL/S sub-chart, 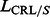 (rounded to an integer) is sufficient [16], [41]



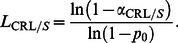
(13)Here, 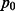 is the in-control probability of a non-conforming TBE, *X* on the *T/S* sub-chart and 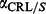 is the Type-I error probability of the CRL/*S* sub-chart. Assume that the process starts at 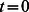, then Figure 1 shows the counts of the CRL, where CRL_1_ = 3, CRL_2_ = 2, CRL_3_ = 4, CRL_4_ = 1.

## Methodology: Development of the Proposed Charts

This section shows the development of the proposed Synth*-T_r_*, GR*-T* and GR*-T_r_* charts. The Synth*-T_r_* chart consists of a *T_r/_S* sub-chart and a CRL*/S* sub-chart while the GR-*T* chart consists of a *T/S* sub-chart and a GCRL/*S* sub-chart. Meanwhile the GR-*T_r_* chart consists of a *T_r_*/*S* sub-chart and a GCRL/*S* sub-chart. Since the GR-*T_r_* chart reduces to the Synth-*T_r_* and GR-*T* charts under some special conditions, the implementation procedure of the GR-*T_r_* chart will be discussed first, followed by the other two charts.

### Implementation

Assume that a homogenous Poisson process having an in-control mean TBE 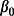 is to be monitored.

#### GR-*T_r_* chart

The implementation of the proposed GR-*T_r_* chart, which integrates the *T_r_/S* and GCRL/*S* sub-charts consists of the following steps:

Step 1: Determine the optimal parameters 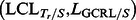, based on the in-control and out-of-control ANOS requirements. The 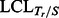 is the lower control limit of the *T_r_* /*S* sub-chart similar to Equation (6), obtained by solving
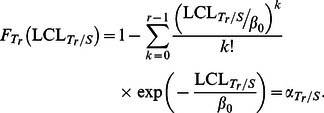
(14)


The 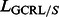 is the lower limit of the GCRL/*S* sub-chart, i.e
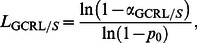
(15)where 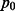 is the in-control probability of non-conforming *Y_r_* on the *T_r_*/*S* sub-chart and 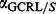 is the Type-I error probability of the GCRL/*S* sub-chart. The calculation of ANOS, based on the Markov chain method is derived in Section 3.2, and the procedure of finding the optimal parameters is given in Section 3.3.

Step 2: Record the TBE observation, *X*. After the *r* ^th^ TBE observation, calculate the sum of the *r* consecutive TBE observations, denoted as 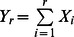.

Step 3: If 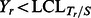, *Y_r_* is considered as non-conforming and the control flow goes to the next step. Otherwise, *Y_r_* is conforming and the control flow returns to step 2.

Step 4: Count the number of conforming *Y_r_* between the current and the last non-conforming *Y_r_*. This counted number is the CRL value of the GCRL/*S* sub-chart. Note that a simple spreadsheet program, like Excel could be used to keep track of the count.

Step 5: If the first CRL is less than or equal to 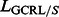, (i.e.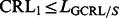), or any two consecutive CRL are both less than or equal to 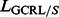 for the first time, such that 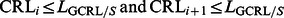, for 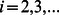, the process is out-of-control and the control flow goes to the next step. Otherwise, the process is in-control and the control flow returns to Step 2.

Step 6: Signal the out-of-control status. Take actions to search and remove the assignable cause(s). Then go back to Step 2.

#### Synth-*T_r_* chart

The implementation of the proposed Synth-*T_r_* chart that combines the *T_r_/S* and CRL/*S* sub-charts comprises the following steps:

Step 1 to Step 4: Determine the optimal parameters 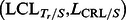, based on the in-control and out-of-control ANOS requirements. Follow the steps in the implementation of the GR-*T_r_* chart, except that the GCRL/*S* sub-chart is replaced by the CRL/*S* sub-chart. In Equation (15), the 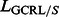 is replaced by the 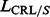 and the 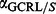 is replaced by the 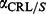.

Step 5: If 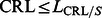, the process is out-of-control and the control flow goes to the next step. Otherwise, the process is in-control and the control flow returns to Step 2.

Step 6: Signal the out-of-control status. Take actions to search and remove the assignable cause(s). Then go back to Step 2.

#### GR-*T* chart

The implementation of the proposed GR-*T* chart which is an integration of the *T/S* and GCRL/*S* sub-charts is similar to that of the GR-*T_r_* chart when *r = *1 (i.e. the GR-*T*_1_ chart).

#### Synth-*T* chart

The implementation of the Synth-*T* chart which is an integration of the *T/S* and CRL/*S* sub-charts is similar to that of the Synth-*T_r_* chart when *r = *1 (i.e. the Synth-*T*_1_ chart).

### Performance Evaluation Based on ANOS Using the Markov Chain Approach

The performance of a TBE control chart is measured by the average run length (ARL) of the chart, which is defined as the average number of TBE points plotted on the chart before a TBE point indicates an out-of-control signal. Based on this definition, the ARL is appropriate to be used for evaluating the performance of the exponential-type chart, i.e. the *T* chart. However, it is not valid to use the ARL while making a comparative study between the exponential-type chart and the Erlang-type chart, i.e. the *T_r_* chart. This is because the exponential-type chart will signal an out-of-control when a single TBE point falls beyond the control limit. However, the Erlang chart requires that the sum of *r* consecutive TBEs is beyond the control limit, in order for the chart to signal. This means that each plotted point on the Erlang chart is not consisting of a single TBE observation. The number of TBE observations to plot a point on the *T* and *T_r_* chart varies. For example, a *T*_3_ chart requires three (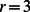) consecutive TBE observations in order to plot a point on the chart. In contrast, the *T* chart only needs a single TBE observation in plotting a point on the chart.

To overcome this problem, we compute the average number of TBEs observed to signal (ANOS) an out-of-control. The relationship between ANOS and ARL is expressed as
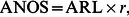
(16)where *r* is the number of cumulative TBEs before a point is plotted. An exponential-type chart, such as the *T* chart, has 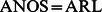 as 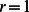. The relationship between the average time to signal (ATS), which is the average time required for a control chart to signal an out-of-control and the ANOS can be expressed as



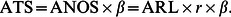
(17)The performance evaluation of the TBE control chart using the ATS and ANOS is similar; the only difference is on their interpretation. However, we prefer to use ANOS over ATS in evaluating the performance of a cumulative quantity control (CQC) chart, like the TBE chart. The ANOS interpretation based on the number of observations is more general and direct compared to the ATS. When the cumulative quantity is measured by say, length, volume, weight, power and so on (other than time) [2], the ATS cannot be relied upon as these quantities are not measured in terms of time. However, ANOS can be used irrespective of whether the quantity is measured in terms of time, length, volume, weight, power, etc.

In this paper, a Markov chain method given in Davis & Woodall [42] and Gadre & Rattihalli [49] is adopted in studying the zero- and steady-state ANOS performance of the Synth-*T* chart proposed by Scariano & Calzada [36], as well as the proposed Synth-*T_r_* chart, GR-*T* chart and GR-*T_r_* chart.

#### Synth-*T_r_* chart

Let 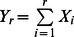 be the sum of *r* TBEs observed. Suppose that each *Y_r_* can be classified as either “0” (conforming, i.e. 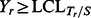) or “1” (non-conforming, i.e. 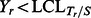). Define
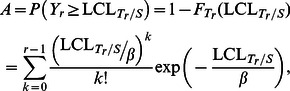
(18)and 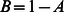. Consider the case when the lower limit of the CRL/*S* sub-chart, 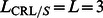. The Synth-*T_r_* chart will signal if 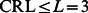. The Markov chain that represents this situation has the following transition probability matrix (TPM) [42]:



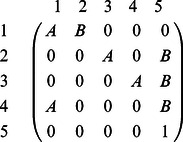
(19)There are a total of four non-absorbing states (Table 1) with an absorbing state (state 5) in the TPM. A 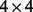 matrix R of the non-absorbing states is obtained after the last row and column of the TPM in Equation (19) is removed. In general, the matrix R of the Synth-*T_r_* chart has 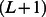 non-absorbing states as stated below:

**Table 1:** The non-absorbing states of the Synth-*T_r_* chart in the Markov chain when *L = *3.

State Number	Non-absorbing State
1	000
2	0001 (initial state)
3	00010
4	000100

1. A sequence of *L* zeros, such as State 1 in Table 1 when 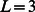.
2. The sequence of *L* zeros in (1) is followed by 1 and further added by at most (*L−*1) zeros. There are *L* such sequences, such as States 2, 3, and 4 in Table 1.

The general matrix R of non-absorbing states will be a 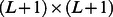 matrix, which is constructed based on the 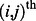 entry of the TPM as follows [49]: 
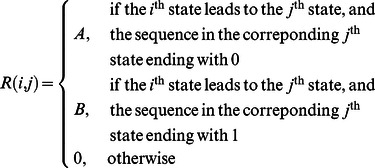
(20)


The zero-state ANOS of the Synth-*T_r_* chart is
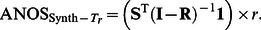
(21)


Here, 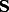 is the 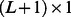 column vector of initial probabilities, “1” for the initial state and “0” for the rest of the cases and 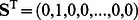 in this synthetic chart; 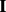 is the identity matrix of size 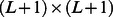; 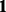 is a column vector of order 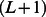 having all elements unity; **R** is a 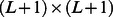 matrix of non-absorbing states and *r* is the value corresponding to the Synth-*T_r_* chart. When the effect of the head start has faded away, the steady-state performance is an important measure. The steady-state ANOS of the Synth-*T_r_* chart is given by
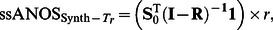
(22)where the matrix 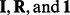 are as defined before. Here, 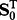 is the 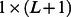 steady-state probability row vector with the stationary probabilities of being in each non-absorbing state. In Equation (22), 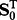 is obtained by solving the system of linear equations of 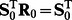 subject to 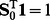, where 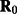 is an adjusted version of 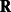 which is obtained from 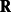 after dividing each element by the corresponding row sum [52]. For example, if 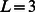,
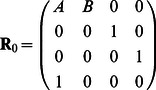
(23)


#### Synth-T chart

It is easy to obtain the zero- and steady-state ANOS of the Synth-*T* chart by just letting 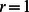 in the procedure for the Synth-*T_r_* chart.

#### GR-*T_r_* chart

Suppose that each 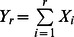 can be classified as either “0” (conforming, i.e. 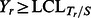) or “1” (non-conforming, i.e. 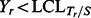). Define 
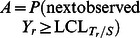
, i.e. similar to Equation (18), and 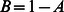. Consider the case when the lower limit of the GCRL/S sub-chart, 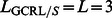. The GR-*T_r_* chart will signal if either the first or two consecutive 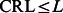 for the first time, such that 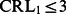 or 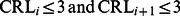, for 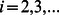. The Markov chain that represents this situation has the following transition probability matrix (TPM) [49]:
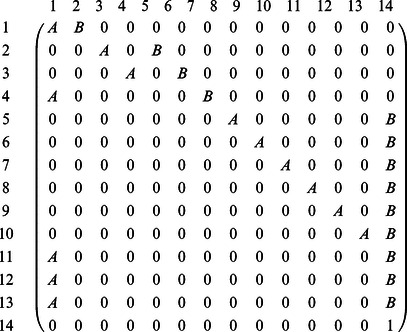
(24)


There are 13 non-absorbing states (Table 2) with an absorbing state (state 14). A 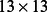 matrix R of non-absorbing states is obtained after the last row and column of the TPM in Equation (24) is removed. In general, the matrix R in the TPM of the GR-*T_r_* chart with a value of *L* has non-absorbing states as follows [49]:

**Table 2:** The non-absorbing states of the GR-*T_r_* chart in the Markov chain when *L = *3.

StateNumber	Non-absorbingState	StateNumber	Non-absorbingState
1	000	8	000110
2	0001	9	0001010
3	00010	10	00010010
4	000100	11	0001100
5	00011 (initial state)	12	00010100
6	000101	13	000100100
7	0001001

1. A sequence of *L* zeros, such as state 1 in Table 2 when 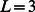.
2. The sequence of *L* zeros in (1) is followed by 1 and is further added by at most (*L*-1) zeros. There are a total of *L* sequences, such as states 2, 3 and 4 in Table 2.
3. Each of the sequences in (2) is followed by 1 and is further added by a sequence of at most 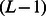 zeros. The total number of such sequences is 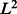, such as states 5 to 13 in Table 2.

Thus, the GR-*T_r_* chart has a square matrix **R** of order.
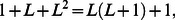
(25)for which the 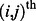 element of **R** is as defined in Equation (20).

Similar to Equation (21), the zero-state ANOS of the GR-*T_r_* chart can be computed by
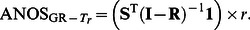
(26)


Here, the matrix **R** is of size 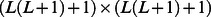; 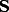 is a 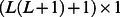 column vector of the initial probabilities such that 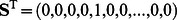; 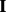 is the identity matrix of size 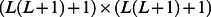; 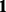 is a column vector of order 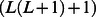 having all elements unity; and *r* is the value corresponding to the GR-*T_r_* chart. Note that the initial state is state 5 when 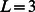 (Table 2). Meanwhile, the steady-state ANOS of the GR-*T_r_* chart can be computed by substituting the matrix **R** of the GR-*T_r_* chart into Equation (22), which gives
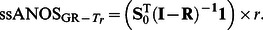
(27)


Here, the matrix 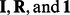 are as defined in the zero-state ANOS of the GR-*T_r_* chart while 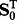 is a 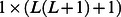 steady-state row vector. 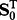 is obtained based on the corresponding 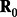, which is an adjusted version of matrix **R** with size 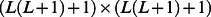.

#### GR-*T* chart

The zero- and steady-state ANOS performance of the GR-*T* chart can be obtained by letting 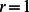 in the Markov chain procedure of the GR-*T_r_* chart, i.e.
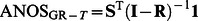
(28)

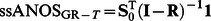
(29)


### Optimal Design Based on ANOS

The optimal design procedure for the proposed charts, based on the zero- and steady-state ANOS described in this section is similar to the design presented in Wu and Spedding [41]. To design the proposed Synth-*T_r_*, GR-*T* and GR-*T_r_* charts, two optimal parameters (*L,* LCL) have to be selected. The objective function to be minimized is the out-of-control ANOS(∂_opt_) with an optimal shift size (which is considered large enough to seriously impair the process quality)
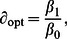
(30)subject to a specified in-control ANOS (ANOS_0_) or an acceptable overall Type-I error probability of 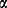. The design procedure is summarized using the following steps:

1. Given an in-control mean TBE 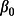, specify the desired ANOS_0_ and 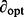.
2. Initialize 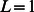.
3. Let 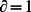 (process is in-control when 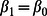).
4. Obtain LCL of (i) Synth-*T_r_* chart using Equations (21) and (22), (ii) GR-*T_r_* chart using Equations (26) and (27), or (iii) GR-*T* chart using Equations (28) and (29), for zero- and steady-state ANOSs, respectively.
5. Let 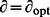, calculate ANOS(∂_opt_) from the current LCL and *L* values.
6. If the current 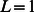, or the ANOS(∂_opt_) value of the current *L* (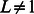) is smaller compared to that of (*L*–1), increase *L* by one and return to Step 4. Otherwise, go to Step 7.
7. Choose the current LCL and *L* values that give the smallest ANOS(∂_opt_) value as the optimal parameters of the proposed chart.
8. Use the optimal LCL in Step 7 as the lower control limit of the *T/S* sub-chart or *T_r_/S* sub-chart. And use the optimal *L* as the lower limit of the CRL/*S* sub-chart or GCRL/*S* sub-chart, depending on which proposed chart is considered.

### Optimization Program

Based on the optimization procedure mentioned in Section 3.3, two Mathematica programs are written to compute the optimal parameters *L* and LCL, for the zero- and steady-state ANOS, respectively, for each of the proposed charts. First, the user has to input the desired values of ANOS_0_, ∂_opt_ and 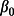. Since these programs are designed to run iteratively, the user needs to input the additional information on *L*_max_, for the program to run until a desired maximum value of *L*. The *L*_max_ in this study is set to be 50. However, to reduce computation time, especially when *r* is large (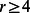), the user may use a smaller *L*_max_ to get the same results. After the desired ANOS_0_ and ∂_opt_ are input, running the program will give a set of results comprising the parameters (*L*, LCL), for 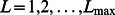, together with the corresponding ANOS(∂_opt_). The programs will also identify the optimal parameters (*L*, LCL) that give the smallest value of ANOS(∂_opt_). After the optimal parameters are obtained, the user has to follow the steps explained in Section 3.1 to implement the optimal Synth-*T_r_*, GR-*T* and GR-*T_r_* charts. The optimal parameters of the Synth-*T* chart can also be obtained using the optimization programs for the Synth-*T_r_* chart by letting 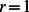.

Additional Mathematica programs are written to compute the optimal parameters 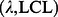 of the EWMA-*T* chart and the parameters of the *T* chart and *T_r_* chart. The Mathematica programs for all the charts considered in this paper can be requested from the first author.

## Results and Discussion: The ANOS Performance of the Optimal Synth-*T_r_*, GR-*T* and GR-*T_r_* Charts

Without loss of generality and for simplicity, consider the case 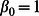. Then the mean TBE 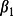 has a decreasing shift when 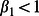 or 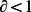. The optimization parameters of the Synth-*T*, Synth-*T_r_*, GR-*T* and GR-*T_r_* charts together with the corresponding zero- and steady-state ANOS(∂_opt_)s are computed using the optimization programs described in Section 3.4. Both the zero- and steady-state ANOS(∂_opt_)s together with the optimal parameters (*L*, LCL) of the proposed charts (Synth-*T_r_*, GR-*T* and GR-*T_r_* charts, for *r* = 2, 3,4, 5) are displayed in Table 3, based on 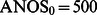 and optimal shift size 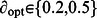}. For the sake of comparison, the ANOS(∂_opt_)s of the *T*, *T_r_* (*r* = 2, 3, 4), Synth-*T* and EWMA-*T* charts are computed. The optimal parameters 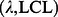 of the EWMA-*T* chart are also computed. The results are shown in Table 3.

**Table 3:** The zero- and steady-state ANOS(∂_opt_)s together with optimal parameters of the TBE charts based on ∂_opt* = *_0.2, 0.5 and ANOS_0_ = 500.

Mode	∂∂∂_opt_	*T*(LCL)	*T*_2_(LCL)	*T*_3_(LCL)	*T*_4_(LCL)	EWMA-*T*(λ, LCL)
Zero-state	0.2	100.501(0.0020)	25.534(0.0902)	11.082(0.3610)	7.439(0.7710)	6.857(0.35, 0.2377)
	0.5	250.500(0.0020)	133.440(0.0920)	81.361(0.3610)	56.319(0.7710)	18.649(0.1, 0.5450)
Steady-state	0.2	100.501(0.0020)	25.534(0.0920)	11.082(0.3610)	7.439(0.7710)	6.470(0.35, 0.2373)
	0.5	250.500(0.0020)	133.440(0.0920)	81.361(0.3610)	56.319(0.7710)	**18.227** **(0.1, 0.5439)**
		Synth-*T*(*L*, LCL)	Synth-*T*_2_(*L*, LCL)	Synth-*T*_3_(*L*, LCL)	Synth-*T*_4_(*L*, LCL)	Synth-*T*_5_(*L*, LCL)
Zero-state	0.2	23.965(1, 0.0457)	5.330(2, 0.3358)	3.913(2, 0.8543)	4.276(1, 1.6747)	5.069(1, 2.4325)
	0.5	131.069(1, 0.0457)	50.193(3, 0.3012)	28.001(4, 0.7455)	19.705(3, 1.3939)	15.962(3, 2.0731)
Steady-state	0.2	27.035(1, 0.0467)	7.377(1, 0.4132)	5.743(1, 0.9999)	6.346(1, 1.6999)	7.583(1, 2.4683)
	0.5	136.060(1, 0.0467)	57.936(1, 0.4132)	35.299(2, 0.8726)	26.265(2, 1.5193)	21.987(2, 2.2387)
		GR-*T*(*L*, LCL)	GR-*T*_2_(*L*, LCL)	GR-*T*_3_(*L*, LCL)	GR-*T*_4_(*L*, LCL)	GR-*T*_5_(*L*, LCL)
Zero-state	0.2	8.509(1, 0.1346)	**3.003** **(2, 0.5433)**	3.221(1, 1.4621)	4.042(1, 2.2967)	5.007(1, 3.1777)
	0.5	76.070(1, 0.1346)	26.342(3, 0.4677)	15.350(3, 1.0670)	11.763(3, 1.7647)	**10.370** **(2, 2.7365)**
Steady-state	0.2	12.138(1, 0.1413)	**5.594** **(1, 0.7351)**	6.321(1, 1.5117)	8.055(1, 2.3702)	10.008(1, 3.2758)
	0.5	85.871(1, 0.1413)	37.019(2, 0.5687)	24.572(2, 1.2435)	20.180(2, 2.0126)	18.561(2, 2.8376)

The smaller the ANOS(∂_opt_) value, the better the chart is, in detecting shifts in the mean TBE. From Table 3, the proposed Synth-*T*_r_, GR-*T* and GR-*T_r_* charts have better overall performances than the existing *T*, *T_r_* and Synth-*T* charts, for the two cases of 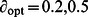. Generally, the EWMA-*T* chart has better performance than the *T* and *T_r_* charts, as well as the Synth-*T* chart (results are consistent with that reported in Scariano and Calzada [36] and Liu et al. [33]).

For both the zero- and steady-state modes, the proposed GR-*T* chart has better performance than the *T*, *T_r_* and Synth-*T* charts, but the EWMA-*T* chart is still superior to the GR-*T* chart. The proposed zero-state Synth-*T_r_* chart surpasses the *T*, *T_r_*, Synth-*T* and GR-*T* charts for both cases of 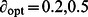 and the EWMA-*T* chart only when 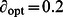. The steady-state ANOS performance of the Synth-*T_r_* chart is quite similar to that of the EWMA-*T* chart in the case of 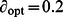, but the former is inferior to the latter in the case of 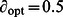. Overall, the zero-state GR-*T_r_* chart is the best chart for detecting the optimal mean shift, especially when 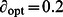. The superiority of the proposed charts in comparison with the EWMA-*T* chart is more obvious for the case of 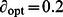.

Specifically, if 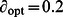, the zero-state GR-*T*_2_ chart with optimal parameters 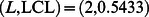 and the corresponding 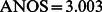 is the most effective chart for detecting the TBE shift of 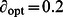. While for 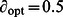, the zero-state GR-*T*_5_ chart with optimal parameters 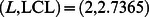 has the smallest ANOS of 10.370 among all the charts. Similar to the zero-state mode, the steady-state GR-*T_2_* chart with optimal parameters 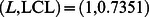 is the best in detecting 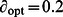. However, when 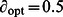, the steady-state ANOS (18.227) of the EWMA-*T* chart is only slightly smaller than that of the GR-*T*_5_ 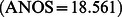 chart. As the difference is negligible, both charts have quite similar performance. For both the zero- and steady-state cases, the GR-*T_r_* chart with a smaller *r* is quicker to detect a larger optimal shift, such as 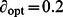 (as compared to 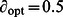) as the number of TBE observations needed increases with *r.* However, the GR-*T_r_* chart with a larger *r* performs better when 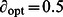 (smaller optimal shift).

For an optimal design based on 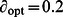 and 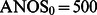, Table 4 displays the computed zero- and steady-state ANOS for the EWMA-*T*, Synth-*T*, Synth-*T_r_*, GR-*T* and GR-*T_r_* charts, for various sizes of mean TBE shifts, 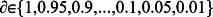. Table 4 shows that for the Synth-*T*, Synth-*T_r_*, GR-*T* and GR-*T_r_* charts, the performance of the zero-state ANOS is better than that of the steady-state for all shifts. However, the EWMA-*T* chart has the same performance for the zero- and steady-state modes. Overall, the proposed Synth-*T_r_*, GR-*T* and GR-*T_r_* charts are superior to the Synth-*T* chart for all shifts. The GR-*T_r_* chart performs better than its Synth-*T_r_* counterpart, while the GR-*T* chart has the poorest performance among the three proposed charts.

**Table 4:** Zero- and steady-state ANOS based on ANOS_0_ = 500 and ∂_opt_ = 0.2.

∂∂∂	EWMA-*T*	Synth-*T*	Synth-*T*_2_	Synth-*T*_3_	Synth-*T*_4_	Synth-*T*_5_	GR-*T*	GR-*T*_2_	GR-*T*_3_	GR-*T*_4_	GR-*T*_5_
	Zero-state										
1.00	500.142	500.047	500.445	500.080	500.012	500.117	500.686	500.033	500.089	500.132	500.113
0.95	380.594	453.339	417.969	393.463	**378.412**	**364.921**	433.756	391.899	**369.331**	**351.356**	**337.346**
0.90	288.184	407.954	346.214	306.793	284.046	264.441	373.082	304.278	270.501	245.336	226.848
0.85	217.234	364.965	284.201	236.930	211.429	190.359	318.360	233.892	196.453	170.362	152.275
0.80	163.120	324.369	230.999	181.126	156.042	136.188	269.280	177.878	141.476	117.751	102.219
0.70	91.240	250.359	147.539	102.443	82.873	68.667	186.817	99.365	71.603	55.779	46.539
0.60	50.927	185.925	89.326	55.296	42.691	34.310	123.233	52.763	35.246	26.537	22.044
0.50	28.786	131.069	50.611	**28.467**	21.578	17.436	76.070	**26.544**	17.118	13.110	11.403
0.40	16.806	85.789	26.398	14.114	11.032	9.495	42.866	12.705	8.492	7.144	6.925
0.30	10.358	50.087	12.457	7.008	6.135	6.086	21.164	5.948	4.647	4.715	5.310
0.25	8.347	35.828	**8.226**	5.098	4.926	5.367	13.859	4.140	3.719	4.232	5.070
0.20	6.857	23.965	5.330	3.912	4.276	5.069	8.509	3.003	3.221	4.041	5.007
0.15	5.742	14.498	3.468	3.270	4.035	5.003	**4.811**	2.353	3.031	4.002	5.000
0.10	4.888	7.432	2.413	3.027	4.000	5.000	2.471	2.061	3.000	4.000	5.000
0.05	4.196	**2.786**	2.019	3.000	4.000	5.000	1.234	2.000	3.000	4.000	5.000
0.01	4.000	1.021	2.000	3.000	4.000	5.000	1.002	2.000	3.000	4.000	5.000
	Steady-state										
1.00	500.644	501.323	500.377	500.042	500.022	500.014	500.383	500.151	500.018	500.125	500.100
0.95	380.462	454.478	421.199	399.132	383.102	**370.577**	436.566	400.655	**378.455**	**362.892**	**350.943**
0.90	287.641	409.926	351.923	316.178	291.603	273.319	378.429	318.586	284.838	262.601	246.464
0.85	216.439	367.666	291.676	248.474	220.505	200.703	325.698	251.358	213.204	189.652	173.451
0.80	162.190	327.700	239.623	193.638	165.667	146.825	278.125	196.690	158.749	136.823	122.516
0.70	90.258	254.646	156.976	114.503	91.806	77.985	197.346	117.294	86.776	71.350	62.314
0.60	50.038	190.765	98.140	65.198	**49.837**	41.431	133.968	67.332	46.785	37.786	33.177
0.50	28.037	136.060	57.940	35.720	26.795	22.520	85.871	37.114	25.193	20.873	19.212
0.40	16.196	90.531	31.852	18.937	14.658	13.106	50.935	19.677	13.946	12.636	12.827
0.30	9.870	54.184	16.048	9.977	8.698	8.867	27.052	10.213	8.466	9.052	10.438
0.25	7.912	39.458	10.989	**7.404**	7.172	7.957	18.603	**7.437**	7.073	8.330	10.093
0.20	6.470	27.035	7.377	5.743	6.346	7.583	12.138	5.594	6.321	8.055	10.008
0.15	5.401	16.921	**4.944**	4.832	6.042	7.504	7.411	4.508	6.042	8.002	10.000
0.10	4.603	9.135	3.512	4.523	6.000	7.500	**4.194**	4.058	6.001	8.000	10.000
0.05	4.015	**3.739**	3.013	4.500	6.000	7.500	2.353	4.000	6.000	8.000	10.000
0.01	3.669	1.526	3.000	4.500	6.000	7.500	2.000	4.000	6.000	8.000	10.000

As compared to the EWMA-*T* chart, in the zero-state mode, the proposed Synth-*T*_4_ and GR-*T_r_* (for *r = *3 and 4) charts outperform the EWMA-*T* chart for all shifts. The ANOSs of the Synth-*T*_5_ and GR-*T*_5_ charts are smaller than that of the EWMA-*T* chart for 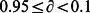; however, when the shifts are very large 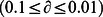, the EWMA-*T* chart slightly outperforms the Synth-*T*_5_ and GR-*T*_5_ charts. Meanwhile, the Synth-*T_r_* (*r* = 2 and 3) and GR-*T*_2_ charts are more sensitive than the EWMA-*T* chart for detecting large shifts, but not for small and moderate shifts. Although the GR-*T* chart is superior to the Synth-*T* chart, but the former is better than the EWMA-*T* chart only for detecting large shifts.

In the steady-state mode, the Synth-*T*_5_ and GR-*T_r_* (*r = *3, 4 and 5) charts always perform better than the EWMA-*T* chart, for detecting small to certain degrees of large shifts. The Synth-*T*_4_ chart is more sensitive than the EWMA-*T* chart for detecting shifts 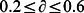. In addition, the Synth-*T*_3_ and GR-*T*_2_ charts are superior to the EWMA-*T* chart for 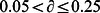. Meanwhile, the ANOSs of the steady-state Synth-*T*_2_ and GR-*T* charts are always greater than that of the EWMA-*T* chart, except when the shift is large.

For the zero-state case, we recommend using the Synth-*T*_2_ chart, GR-*T*_2_ chart or even the GR-*T* chart, instead of the EWMA-*T* chart if the detection of large shifts is desirable. For detecting moderate shifts, the Synth-*T_r_* (*r* = 3, 4 or 5) chart or GR-*T_r_* (*r* = 2, 3, 4 or 5) chart is recommended while for detecting small shifts, the Synth-*T_r_* (*r* = 4 or 5) chart or GR-*T_r_* (*r* = 3, 4 or 5) chart is recommended. For the steady-state case, we recommend the use of the Synth-*T*_2_ or GR-*T* chart for detecting large shifts. However, when detecting shifts 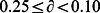 is a concern, the Synth-*T*_3_ and GR-*T*_2_ charts are recommended. For detecting shifts 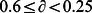, one can consider the Synth-*T_r_* (*r* = 4 or 5) or GR-*T_r_* (*r* = 3, 4 or 5) chart. Furthermore, if only small shifts are to be detected quickly, the Synth-*T*_5_ chart or GR-*T_r_* (*r* = 3, 4 or 5) chart can be considered. Among these charts, the GR-*T*_5_ chart is the most sensitive for detecting small shifts.

### Illustrative Examples

To show the implementation and application of the proposed control charts on different events monitoring, we use a real data set on coal mining accidents and two simulated data set, each on time between component failures and waiting times of outpatients in a hospital.

#### Example I

The coal mining accidents data set in Jarrett [9] is taken to illustrate the construction of the proposed GR-*T_3_* chart. The data set can be obtained from http://www.stat.sc.edu/rsrch/gasp/poicha/mining.dat. The data consist of the time intervals in days between successive coal mining accidents which involve more than ten men killed during the period 1851 to 1962 in Great Britain. The time between accidents, *X* has been shown to be exponentially distributed. The mean of the time between accidents is estimated based on the first 50 observations (similar to [29]) as
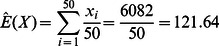



**Figure 2:**
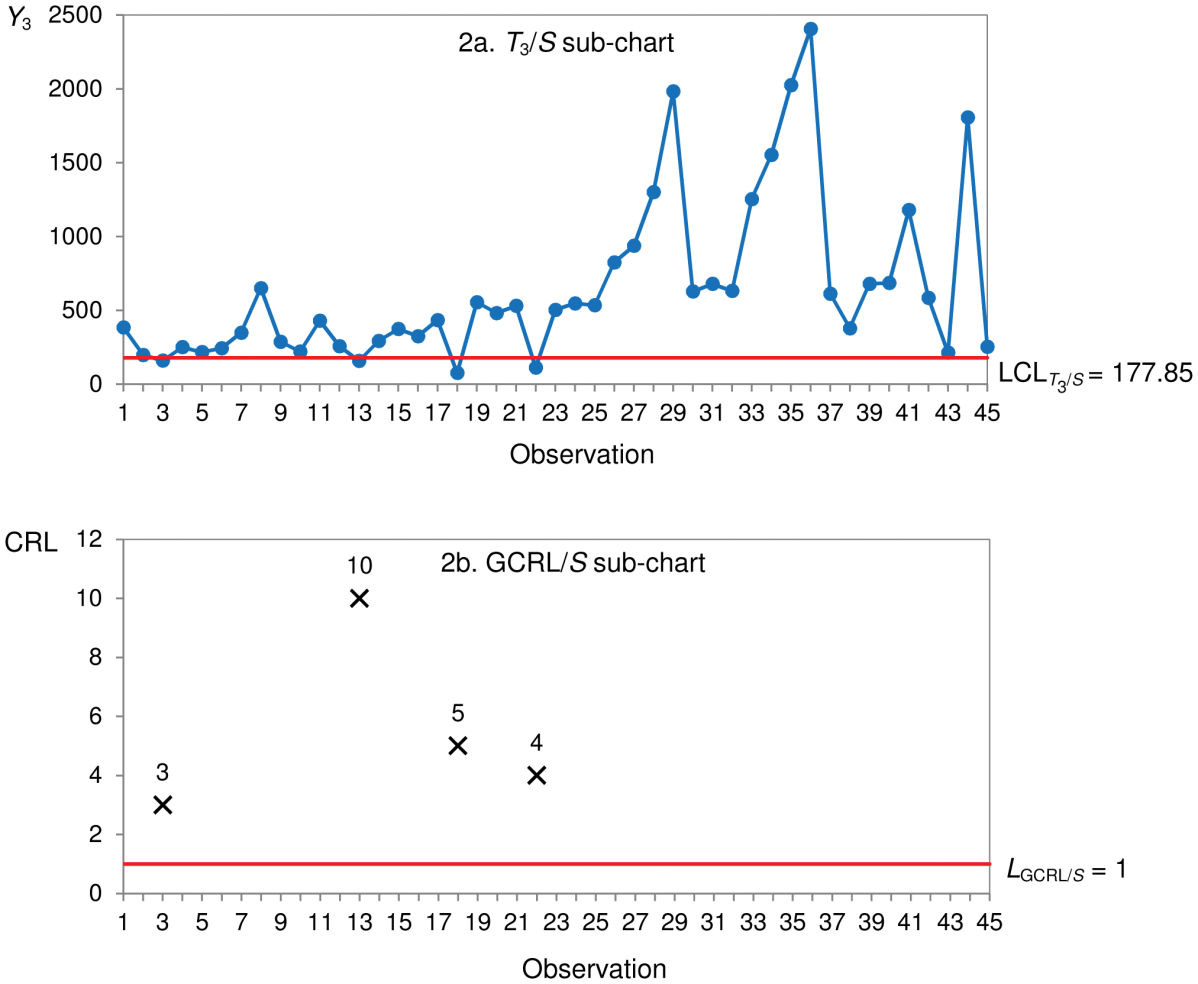
GR-*T*_3_ chart.

Thus, the in-control mean time between accidents is 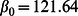. As 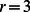, let *Y*_3_ denote the time until the 3^rd^ accident. The GR-*T_3_* chart (consists of a *T_3_/S* sub-chart and a GCRL*/S* sub-chart) to monitor the coal mining accidents data is shown in Figure 2.

For the GR-*T*_3_ chart to be optimal in detecting 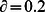 with an in-control zero-state 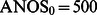, the chart parameters are obtained from Table 3. When 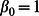, the optimal parameters for the zero-state GR-*T*_3_ chart are 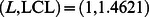. Thus, the *T*_3_ */S* sub-chart and GCRL/*S* sub-chart have lower control limits, 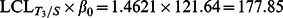 and 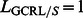 respectively, as optimal parameters. In Figure 2a, the *T*_3_ */S* sub-chart having four observations *Y*_3_ below 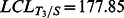 indicates the occurrence of four non-conforming *Y*_3_ observations. Thus, the GCRL/S sub-chart has CRL_1_ = 3, CRL_2_ = 10, CRL_3_ = 5 and CRL_4_ = 4 (Figure 2b). The GR-*T*_3_ chart does not show any out-of-control signal as none of the CRL values is less than or equal to 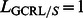. This indicates that the coal mining accidents events are in-control.

#### Example II

**Figure 3:**
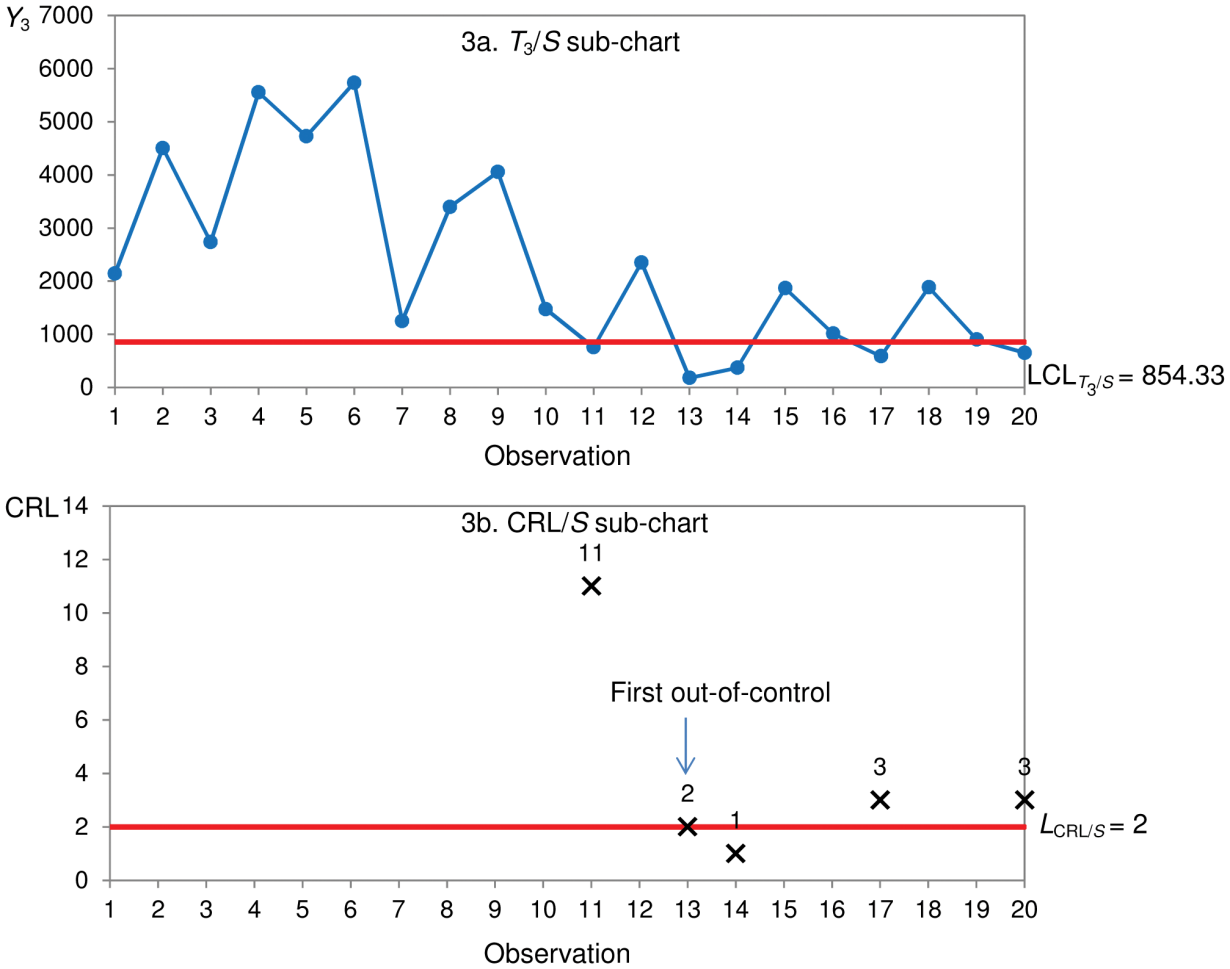
Synth-*T*_3_ chart.

A set of simulated components failure data is taken from Xie et al. [5]. The data consist of 60 observations on the time between components failures. The first 30 observations were simulated from an in-control mean time between failures 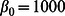, and the remaining 30 observations were simulated from an out-of-control mean time between failures 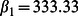. To construct a Synth-*T*_3_ chart, the cumulative time of every three consecutive time between failure observations, *Y*_3_ was recorded [5]. The optimal parameters 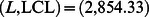 of the Synth-*T*_3_ chart in optimally detecting a mean shift of 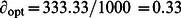, based on 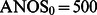 are computed using the optimization program mentioned in Section 3.4. The Synth-*T*_3_ chart with 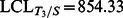 for the *T*_3_/*S* sub-chart and 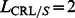 for the CRL/*S* sub-chart is constructed in Figure 3.

An observation *Y*_3_ below 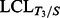 is an indication of a non-conforming observation (Figure 3a). Thus, the CRL/*S* sub-chart (Figure 3b) has CRL_1_ = 11, CRL_2_ = 2, CRL_3_ = 1, CRL_4_ = 3 and CRL_5_ = 3. Since 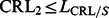 and 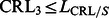 out-of-control time between failures of the components are detected at observations 13 and 14. The Synth-*T*_3_ shows that the first out-of-control signal occurs at observation 13 (Figure 3b), which corresponds to the 39^th^ (13×3) failure.

#### Example III

**Figure 4:**
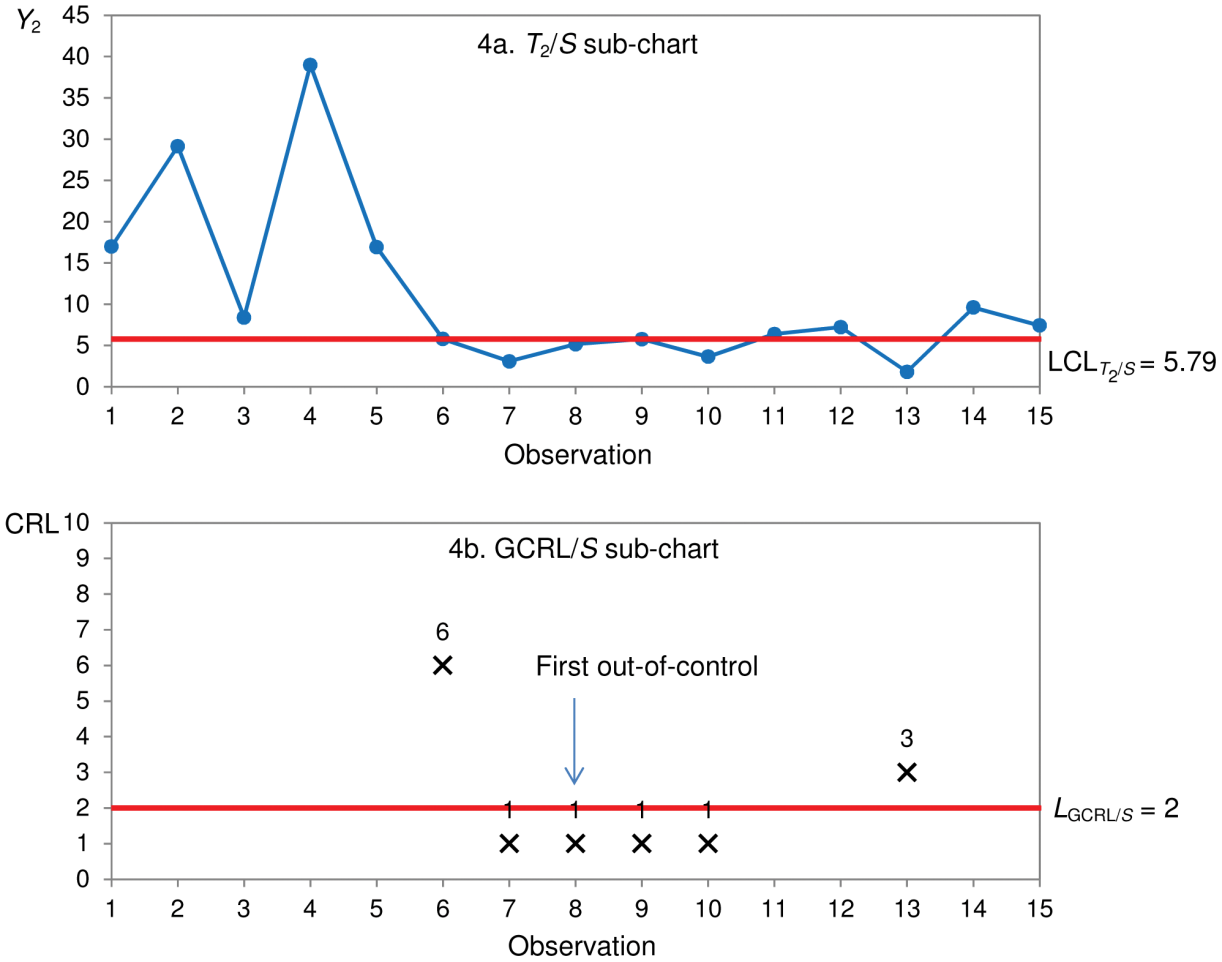
GR-*T*_2_ chart.

Assume that the waiting time of outpatients in a hospital follows the exponential distribution with the mean waiting time of ten minutes (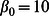). To reduce the mean waiting time from ten minutes to three minutes (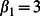), a control chart is used to monitor the improvement in hospital services. The simulated waiting time data given in Xie et al. [8] are considered. The first 10 observations were simulated with 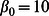 and the last 20 observations with 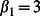. In order to construct a GR-*T*_2_ chart, the sum of every two waiting time observations, *Y*_2_ is recorded. By setting 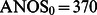 (similar to Xie et al. [8]), the GR-*T_2_* chart having optimal parameters 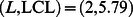 is designed to optimally detect a TBE mean shift, 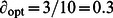. A GR-*T*_2_ chart which consists of a *T*_2_ */S* sub-chart 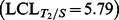 and a GCRL/*S* sub-chart 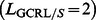 is displayed in Figure 4.

An observation *Y*_2_ falling below 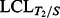 in the *T*_2_ */S* sub-chart (Figure 4a) is non-conforming. In the GCRL/S sub-chart (Figure 4b), CRL_1_ = 6, CRL_2_ = 1, CRL_3_ = 1, CRL_4_ = 1, CRL_5_ = 1 and CRL_6_ = 3. Since 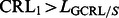, no out-of control signal is given at observation 6. The GR-*T*_2_ chart issues the first out-of-control signal at observation 8 as 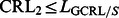 and 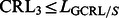 (Figure 4b), which corresponds to the 16^th^ (8×2) waiting time.

### Conclusion

Among the existing *T*, *T_r_*, Synth-*T* and EWMA-*T* charts, the *T* chart is the least sensitive in the detection of all sizes of shifts. The *T_r_* and Synth-*T* charts are better than the EWMA-*T* chart only for detecting large mean TBE shifts. This study proposes three charts, i.e. the Synth-*T_r_*, GR-*T* and GR-*T_r_* charts, to increase the sensitivity of TBE type charts toward mean TBE shifts. The objective of this study is attained as the zero-state Synth-*T_4_* and GR-*T_r_* (*r* = 3 or 4) charts can be used in place of the existing TBE type charts for quicker detection of mean TBE shifts. However, for the steady-state process, the choice between the two best overall charts, namely the existing EWMA-*T* and proposed GR-*T_r_* (r = 3, 4 or 5) charts can be made under different shift intervals, deemed important for a quick detection.

From the results, in general, the superiority of the proposed charts to the existing *T*, *T_r_*, Synth-*T* and EWMA-*T* charts is more obvious when the charts are optimally designed for detecting small and moderate shifts. The zero- and steady-state cases of the proposed Synth-*T_r_*, GR-*T* and GR-*T_r_* charts outperform that of the *T*, *T_r_* and Synth-*T* charts in detecting all sizes of mean shifts. In comparison with the EWMA-*T* chart for the zero-state case, the Synth-*T*_4_ and GR-*T_r_* (*r* = 3 or 4) charts are superior toward all shifts, whereas the Synth-*T_r_* (*r* = 2 or 3) and GR-*T*_2_ charts perform better for moderate and large shifts; and the GR-*T* chart is the most effective only for detecting large shifts. For the steady-state process, the Synth-*T*_5_ and GR-*T_r_* (*r* = 3, 4 or 5) charts are more sensitive than the EWMA-*T* chart for detecting small and moderate shifts. The Synth-*T*_4_ chart surpasses the EWMA-*T* chart for moderate shifts. For detecting large shifts, the Synth-*T*_2_ and GR-*T* charts perform best.

Although increasing *r* results in quicker detection of small and moderate mean shifts, but it also reduces the speed of detecting large shifts (and even some moderate shifts). Furthermore, the value of *r* should not be too large (e.g., *r* >5) so that the waiting time for decision making will not be too long, especially for a process with infrequent occurrences of events.

The implementation of the synthetic-type TBE control charts that involves some calculations and procedures will not be a problem to practitioners due to the availability of powerful and advanced computers, as well as user friendly softwares that will perform all the complicated calculations. This enables the control limits of the *T*/S or *T_r_*/*S* sub-chart and CRL/S or GCRL/S sub-chart to be easily computed via the use of a computer.

Research works of the proposed charts with estimated parameters, the robustness study of the charts and the statistical and economic designs of the charts, based on the average number of observations to signal (ANOS) or median number of observations to signal can be carried out in future to increase the competitiveness of the charts and to enhance the charts' practical advantages.

## Supporting Information

Appendix S1 (DOC)
