# Supplementary material for: Synthetic-Type Control Charts for Time-Between-Events Monitoring
Source: PLoS One. 2013 Jun 3;8(6):e65440. doi: 10.1371/journal.pone.0065440 (PMC3670912; doi:10.1371/journal.pone.0065440)
Supplement: Appendix S1 — (DOC) [file pone.0065440.s001.doc]

# Appendix S1

This section explains the Markov chain approach for computing the ANOS of the EWMA-*T* chart, presented in Pehlivan and Testik and Zhang et al. . For a one-sided EWMA-*T* chart with design parameters$\lambda,\text{ LCL}_{E-T}\text{ and }B$, let the interval between the boundary *B* and the $\text{LCL}_{E-T}$, i.e. $\left(\text{LCL}_{E-T},\text{ }B\right)$ of the in-control region be divided into *n* subintervals, each of width $2\delta$ (Figure 5 ) such that

$\delta=\frac{B-\text{LCL}_{E-T}}{2n}$ (A1)

FIGURE 5 HERE

Each subinterval represents a state and there are *n* transient states altogether. Since the TBE observation is always a positive value, the out-of-control region represent the absorbing state, which is the (*n*+1)^th^ state. The statistic $Z_{t}$ can fall in any of the *n* transient states when the process is in-control and fall in the (*n*+1)^th^ absorbing state when the process is out-of-control. The $Z_{t}$ is said to be in a transient state *j* at time *t* when it falls in the *j*^th^ subinterval $\left(B-2\delta j,B-2\delta\left(j-1\right)\right)$ for $j=1,2,...,n.$ The midpoint of *j*^th^ subinterval is represented by

$H_{j}=\frac{B-2\delta j+B-2\delta\left(j-1\right)}{2}=B-2\delta j+\delta=B-\left(2j-1\right)\delta$. (A2)

The run length of the EWMA-*T* chart is defined as the number of steps taken from an initial state of $Z_{0}$ until $Z_{t}$ falls in the absorbing state, which can be determined by a transition probability matrix. Let $P_{ij}$be the transition probability among transient states that $Z_{t}$ transitions from state *i* (for $i=1,2,\ldots,n$ at time $t-1$) to state *j* (for $j=1,2,\ldots,n$ at time *t*). Assume that $Z_{t-1}$ is equal to $H_{i}$ in state *i* at time $t-1$. Since the largest value of *Z_t_* is set as *B*, the largest *Z_t_* always fall in state 1 of interval $\left[B-2\delta,\text{ }B\right)$. Thus, when $j=1$, the transition from state *i* to the 1*^st^* state is given by

$\begin{matrix} P_{i1}=P\left[B-2\delta\leq Z_{t}<B\left|Z_{t-1}=H_{i}\right.\right] \\ =P\left[H_{1}-\delta\leq Z_{t}<B\left|Z_{t-1}=H_{i}\right.\right] \\ =P\left[Z_{t}\geq H_{1}-\delta\left|Z_{t-1}=H_{i}\right.\right] \\ =P\left[\lambda X_{t}+(1-\lambda)Z_{t-1}\geq H_{1}-\delta\left|Z_{t-1}=H_{i}\right.\right] \\ =P\left[X_{t}\geq\frac{H_{1}-\delta-(1-\lambda)H_{i}}{\lambda}\right] \\ =1-F_{T}\left(\frac{H_{1}-\delta-(1-\lambda)H_{i}}{\lambda}\right),i=1,...,n \end{matrix}$ (A3)

The $P_{ij}$ for state $j=2,\text{3,}\ldots,n$ is given as

$\begin{matrix} P_{ij}=P\left[B-2\delta j\leq Z_{t}<B-2\delta\left(j-1\right)\left|Z_{t-1}=H_{i}\right.\right] \\ =P\left[B-2\delta j\leq\lambda X_{t}+(1-\lambda)Z_{t-1}<B-2\delta\left(j-1\right)\left|Z_{t-1}=H_{i}\right.\right] \\ =P\left[B-2\delta j\leq\lambda X_{t}+(1-\lambda)H_{i}<B-2\delta\left(j-1\right)\right] \\ =P\left[\frac{\left(B-2\delta j\right)-(1-\lambda)H_{i}}{\lambda}\leq X_{t}<\frac{\left(B-2\delta j+2\delta\right)-(1-\lambda)H_{i}}{\lambda}\right] \\ =P\left[\frac{H_{j}-\delta-(1-\lambda)H_{i}}{\lambda}\leq X_{t}<\frac{\left(H_{j}+\delta\right)-(1-\lambda)H_{i}}{\lambda}\right] \\ =F_{T}\left(\frac{\left(H_{j}+\delta\right)-(1-\lambda)H_{i}}{\lambda}\right)-F_{T}\left(\frac{H_{j}-\delta-(1-\lambda)H_{i}}{\lambda}\right),i=1,...,n;j=2,...,n \end{matrix}$ (A4)

The zero-state ANOS for the EWMA-*T* chart is

$\text{ANOS}_{\text{EWMA}-T}=\mathbf{S}^{\text{T}}\left(\mathbf{I}-\mathbf{R}\right)^{-1}\mathbf{1}$, (A5)

where **R** is the $n\times n$ transition probability matrix of the transient states with elements$P_{ij}$; **I** is a $n\times n$ identity matrix; **1** is a $n\times1$ row vector of ones and; $\mathbf{S}$ is a $n\times1$ initial probability vector which determines the starting state of the EWMA-*T* chart. Meanwhile, the out-of-control steady-state ANOS for the EWMA-*T* chart is

$\text{ssANOS}_{\text{EWMA}-T}=\mathbf{S}_{0}^{\text{T}}\left(\left(\mathbf{I }-\mathbf{ R}_{1}\right)^{-\mathbf{1}}\mathbf{1}-0.5\right)$, (A6)

where matrix $\mathbf{I}\text{ and vector }\mathbf{1}$ are as defined previously. Here, $\mathbf{R}_{1}$is the out-of-control transition probability matrix of the transient states; $\mathbf{S}_{0}^{\text{T}}$ is the steady-state probability vector, obtained by first normalizing the in-control transition probability matrix $\mathbf{R}$(based on $\partial=1$) and then solving the equation $\mathbf{S}_{0}=\mathbf{R}^{\text{T}}\mathbf{S}_{0}$subject to $\mathbf{1}^{\text{T}}\mathbf{S}_{0}=1$. For an accurate approximation of ANOS*_E-T_* using the Markov chain method, $n=201$ is considered in this study. The ANOS computed have been verified with simulation. The ANOS performance is similar to the ARL performance, for the EWMA-*T* chart as $r=1$.
